# Editorial: New players on the monoaminergic field: relevance to the mental disorders

**DOI:** 10.3389/fphar.2024.1504261

**Published:** 2024-10-14

**Authors:** Eliyahu Dremencov, Daniela Jezova

**Affiliations:** ^1^ Institute of Molecular Physiology and Genetics, Centre of Biosciences, Slovak Academy of Sciences, Bratislava, Slovakia; ^2^ Institute of Experimental Endocrinology, Biomedical Research Center, Slovak Academy of Sciences, Bratislava, Slovakia

**Keywords:** monoamine neurotransmitters, trace amines, endogenous opioids, endocannabinoids, corticosteroids, adenosine, psychedelics, trophic factors

**FIGURE 1:**
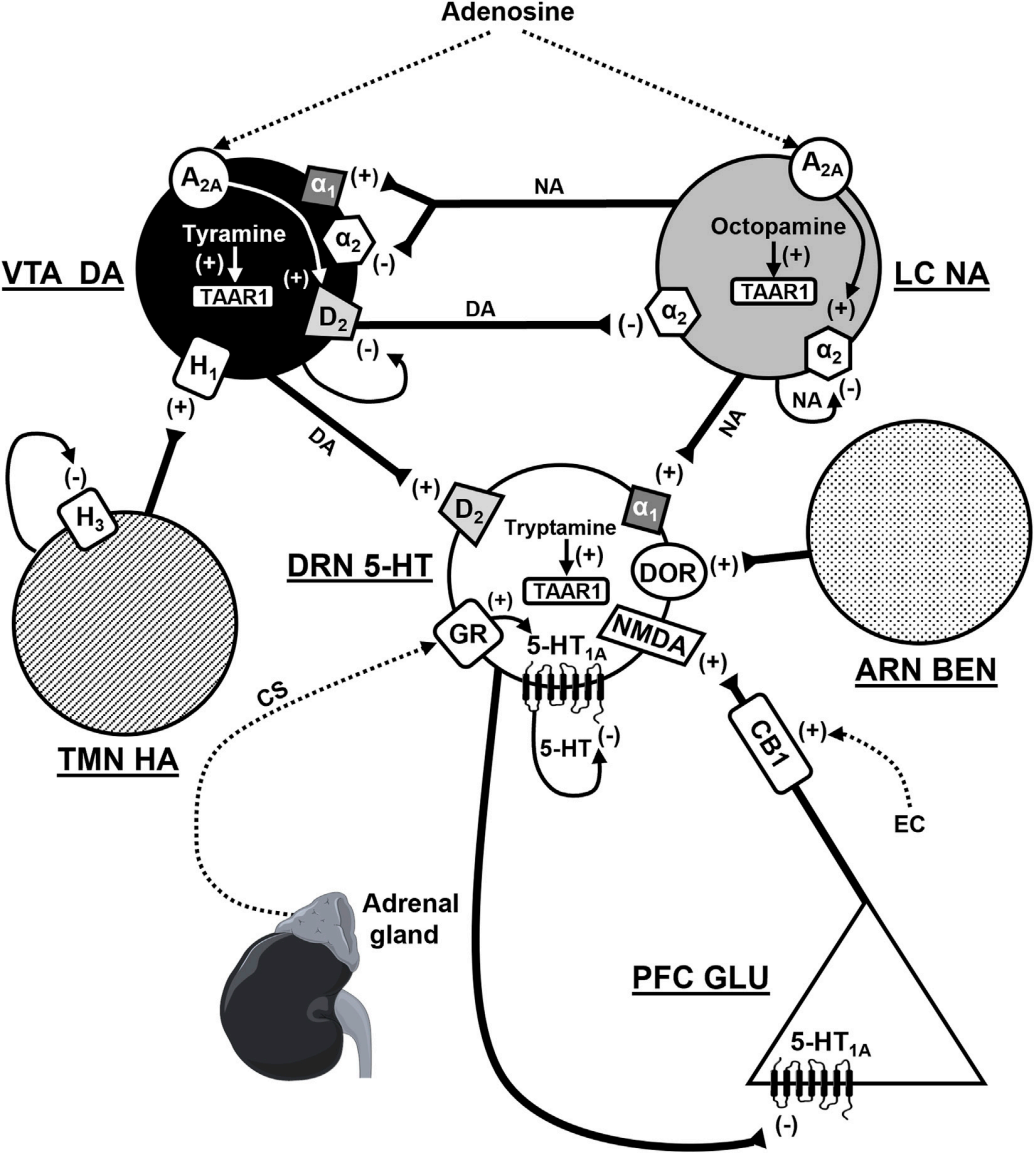
Functional interactions between monoamines and other signaling biomolecules. VTA DA, ventral tegmental area dopamine neuron, LC NA, locus coeruleus noradrenaline neuron, TMN HA, tuberomammillary nucleus histamine neuron, DRN 5-HT, dorsal raphe nucleus serotonin (5-HT) neuron, ARN BEN, arcuate nucleus β-endorphin neuron, PFC GLU, prefrontal cortex glutamate neuron, A_2A_, adenosine-2A receptor, D_2_, dopamine-2 receptor, α_1/2_-alpha-1/2 adrenoceptor, H_1/3_-histamine-1/3 receptor, 5-HT_1A_, serotonin-1A receptor, DOR-δ-opioid receptor, GR, glucocorticoid receptor, NMDA, N-methyl-D-aspartate glutamate receptor, CB1, cannabinoid-1 receptor, TAAR1, trace amine associated receptor 1, CS, corticosteroids, EC, endocannabinoids, +, stimulatory effect, -, inhibitory effect. According to (El Mansari et al., 2010; Dremencov et al., 2017; Liu et al., 2024; Flik et al., 2015; Zhao et al.; Dremencov et al., 2022; Dremencov et al., 2023).

It is well established that monoamines, such as serotonin (5-HT), noradrenaline, and dopamine, are fundamental in emotions and mood regulation. They also play a key role in the pathophysiology and treatment of mood disorders, such as unipolar and bipolar depression, as well as anxiety disorders, such as generalized anxiety, obsessive-compulsive, panic, and post-traumatic stress disorders (Stahl et al., 2014). We may underline three factors that are of critical importance for the proper understanding of the role of monoamines in mood disorders. The first one is the autoregulatory mechanism within the monoaminergic circuits (Blier, 2001). The second one is the multiple cross-interaction between different monoamines (El Mansari et al., 2010). The third factor is the crosstalk between monoamines and other bioactive molecules influencing mood regulation and mental functions in general. Examples of particularly important bioactive molecules are amino acid neurotransmitters, trace amines (biological amines biochemically related to the “classical” monoamines, but present in the brain in trace concentrations, such as tyramine, octopamine, and tryptamine), corticosteroids, adenosine, endogenous opioids, and cannabinoids (Figure 1).

While the auto- and cross-regulatory mechanisms of the central monoaminergic systems have been extensively studied in the last decades, the crosstalk between monoamines and specific other biomolecules involved in mood regulation received lesser attention. This Research Topic of articles was prepared in order to diminish this gap in knowledge and to stimulate future research in this direction.

The task was completed thanks to the international team of researchers, coming from different parts of the world (Asia, America, Europe) and employment sectors (universities, public hospitals and research centers, and industrial enterprises). These researchers contributed their outstanding articles addressing the abovementioned points.

Zhao et al. from the Qingdao University, Qingdao, China, provided a review article on endocannabinoid-monoamine interactions, learning, and memory. Lerer et al. from the Hadassah Medical Center, Jerusalem, Israel, contributed original research on the neurochemical mechanisms underlying putative beneficial effect of psychedelics in mental disorders. Dremencov et al. from the Centre of Biosciences and Jezova et al. from Biomedical Research Center, Slovak Academy of Sciences, Bratislava, Slovakia, shared with us an original research article on functional interactions between monoamines, glutamate, and endogenous opioids, focusing on prenatal effect of the ligands of opioid receptors on monoaminergic and glutamatergic transmission. Daniels et al. from the Institute of Mental Health Research, University of Ottawa, Ottawa, Canada, prepared an original research paper on glutamate-monoamine interactions and their role in the rapid antidepressant effect of ketamine. Malik et al. from the COMSATS University Islamabad, Islamabad, Pakistan, shared with us the results of their research on the interactions between monoamines, adenosine, microelements and steroids, with a practical focus on the putative anxiolytic and antidepressant-like effects of a natural element, diosgenin. Kolaczynska et al. from the University of Basel, Basel, Switzerland, and their co-authors from the Medical University of Vienna, Vienna, Austria (Dino Luethi), ReseaChem GmbH, Burgdorf, Switzerland (Daniel Trachsel), and Roche Innovation Center Basel, F. Hoffmann-La Roche Ltd., Basel, Switzerland (Marius C. Hoener), shared with us their interesting and actual results on psychedelics and “classical” monoamines-trace amines interactions.

The original findings published in the current Research Topic of articles clearly reflect the broadening of the field of classical monoamines towards a complex cocktail of interactive actions of neurotransmitters and other biologically active substances. Of particular importance are the relationships of monoamines with glutamate, endogenous cannabinoids and opioids, adenosine, microelements, glucocorticoids, and trace amines. There is a clear motivation for future research in this important field of neuroscience related to the development, course, and treatment of mental disorders.
